# Lead Levels and Ischemic Heart Disease in a Prospective Study of Middle-Aged and Elderly Men: the VA Normative Aging Study

**DOI:** 10.1289/ehp.9629

**Published:** 2007-02-06

**Authors:** Nitin B. Jain, Vijayalakshmi Potula, Joel Schwartz, Pantel S. Vokonas, David Sparrow, Robert O. Wright, Huiling Nie, Howard Hu

**Affiliations:** 1 Channing Laboratory, Department of Medicine, Brigham and Women’s Hospital and Harvard Medical School, Boston, Massachusetts, USA; 2 Health Investigations Branch, Centers for Disease Control and Prevention, Agency for Toxic Substances and Disease Registry, Atlanta, Georgia, USA; 3 Department of Environmental Health, Harvard School of Public Health, Boston, Massachusetts, USA; 4 Normative Aging Study, VA Boston Healthcare System and Department of Medicine at Boston University School of Medicine, Boston, Massachusetts, USA

**Keywords:** angina, epidemiology, myocardial infarction

## Abstract

**Background:**

Lead exposure has been associated with higher blood pressure, hypertension, electrocardiogram abnormalities, and increased mortality from circulatory causes.

**Objective:**

We assessed the association between bone lead—a more accurate biomarker of chronic lead exposure than blood lead—and risk for future ischemic heart disease (IHD).

**Methods:**

In a prospective cohort study (VA Normative Aging Study), 837 men who underwent blood or bone lead measurements at baseline were followed-up for an ischemic heart disease event between 1 September 1991 and 31 December 2001. IHD was defined as either a diagnosis of myocardial infarction or angina pectoris that was confirmed by a cardiologist. Events of fatal myocardial infarction were assessed from death certificates.

**Results:**

An IHD event occurred in 83 cases (70 nonfatal and 13 fatal). The mean blood, tibia, and patella lead levels were higher in IHD cases than in noncases. In multivariate Cox-proportional hazards models, one standard deviation increase in blood lead level was associated with a 1.27 (95% confidence interval, 1.01–1.59) fold greater risk for ischemic heart disease. Similarly, a one standard deviation increase in patella and tibia lead levels was associated with greater risk for IHD (hazard ratio for patella lead = 1.29; 95% confidence interval, 1.02–1.62).

**Conclusions:**

Men with increased blood and bone lead levels were at increased risk for future IHD. Although the pathogenesis of IHD is multifactorial, lead exposure may be one of the risk factors.

Although blood lead levels in the United States and other industrialized nations have declined over the past decades, pockets of high lead exposure and widespread low-level lead exposures still persist (Pirkle et al. 1994). Moreover, a substantial proportion of the population has had higher lead exposure from leaded gasoline and other sources such as soldered cans, paints, and tap water in the past (Pirkle et al. 1994). The long-term consequences of lead exposure include circulatory diseases, kidney diseases, and neurologic disorders (Cheng et al. 2001; Harlan 1988; Hertz-Picciotto and Croft 1993; Hu et al. 1996a; Kopp et al. 1988; Lin et al. 2003; Martin et al. 2006; Moller and Kristensen 1992; Nash et al. 2003; Pirkle et al. 1985; Schwartz 1991, 1995; Tsaih et al. 2004).

Lead exposure has been associated with increased blood pressure and hypertension in cross-sectional as well as longitudinal studies (Cheng et al. 2001; Harlan 1988; Hertz-Picciotto and Croft 1993; Hu et al. 1996a; Kopp et al. 1988; Martin et al. 2006; Moller and Kristensen 1992; Nash et al. 2003; Pirkle et al. 1985; Schwartz 1991, 1995). More recently, there is evidence of increased mortality from circulatory causes in individuals with blood lead levels of 20–29 μg/dL in the past (Lustberg and Silbergeld 2002). However, the association between lead levels and risk for future ischemic heart disease (IHD) after controlling for potential confounders has not been established. The three previous reports on the possible association between lead levels and cardiovascular disease found no such evidence (Kromhout 1988; Moller and Kristensen 1992; Pocock et al. 1988). These reports used blood lead level as a biomarker for lead exposure, which is now known to poorly reflect the cumulative internal dose of lead. Instead, more recently, bone lead has become the biologic marker of choice to assess long-term lead exposure (Landrigan 1991). With the development of *in vivo* K X-ray fluorescence (KXRF), it is now possible to safely and rapidly measure bone lead in large-scale epidemiologic studies (Landrigan and Todd 1994).

The objective of our study was to assess the relationship of bone and blood lead levels with risk for IHD (fatal and nonfatal) in a longitudinal cohort of aging men.

## Materials and Methods

### Study population

Participants in our study were from the Normative Aging Study (NAS), a longitudinal study of aging established by the Veterans Administration (now Department of Veterans Affairs) in 1961 (Bell et al. 1972). The study cohort initially consisted of 2,280 community-dwelling men who were health-screened from the Greater Boston area; those with chronic medical conditions such as heart disease, diabetes, cancer, peptic ulcer, gout, recurrent asthma, bronchitis, or sinusitis were excluded. Those with either systolic blood pressure > 140 mm Hg or diastolic blood pressure > 90 mm Hg were also excluded. The men were between 21 and 80 years of age (mean, 42 years) on entry into the cohort. Participants subsequently returned for examinations every 3–5 years during the follow-up period. At each visit, extensive physical examination, laboratory, anthropometric, and questionnaire data were collected.

Measurement of blood lead began in 1988 during each continuing regularly scheduled visit of the participant. Beginning in September 1991, permission was sought from each participant to obtain KXRF bone lead measurements. Consenting individuals reported to the Ambulatory Clinical Research Center of the Brigham and Women’s Hospital in Boston. Of the 1,278 participants seen for their regularly scheduled NAS visits from 1 September 1991 through 31 December 2001, our study included participants who had information on either blood or bone lead level and had at least one follow-up visit in this time frame (*n* = 1,019). The major reason given for nonparticipation in the bone lead study was the inconvenience involved in making a separate visit to our bone lead test facility. After excluding participants with a history of IHD (myocardial infarction or angina) before their year of baseline lead measurement visit, the final data set for analysis included 837 participants. These 837 participants had their baseline lead measurement done during their first scheduled visit after September 1991. Approval for this study was obtained from the Human Research Committees of Brigham and Women’s Hospital and the Department of Veterans Affairs Outpatient Clinic. This study complied with all applicable requirements of the United States (including institutional review board approval), and all participants gave written informed consent before the study.

### History and physical parameters

Each NAS participant reported to the study center in the morning after an overnight fast and abstinence from smoking. At the start of the visit, height and weight were measured with the participant wearing only stockings and undershorts. A complete medical history, including identity and purpose of medications taken daily, was elicited by a physician. A history of physician-diagnosed diabetes mellitus and hypertension since the last visit was also elicited. A participant was considered as having a family history of hypertension if either a parent or a sibling had hypertension. The American Thoracic Society questionnaire (Ferris 1978) was used to assess current smoking and past history of smoking, and the Food Frequency Questionnaire (Ward et al. 1994; Willett et al. 1988) was used to assess alcohol consumption.

The participants were asked about history of heart disease since their last visit. Every report of IHD event was reviewed by a board-certified cardiologist, who was unaware of the participant’s blood and bone lead levels. The criteria for myocardial infarction and angina pectoris were those used in the Framingham Heart Study (Shurtleff 1974). A diagnosis of myocardial infarction was defined by unequivocal electrocardiographic changes (i.e., pathologic Q waves), diagnostic increases in serum glutamic-oxaloacetic transaminase and lactic dehydrogenase, and concurrent chest discomfort consistent with myocardial infarction, or by autopsy. Angina pectoris was diagnosed when the participant reported recurrent chest discomfort that lasted up to 15 min and was distinctly related to exertion and relieved by rest or nitroglycerin. Events of fatal IHD were assessed from death certificates. Regular mailings to NAS participants were used to maintain vital status information, and death certificates were obtained for all decedents.

Immediately after the history was obtained, blood pressure was measured using a standard mercury sphygmomanometer with a 14-cm cuff by a physician. With the subject seated for at least 3 min, systolic blood pressure and fifth-phase diastolic blood pressure were measured in each arm to the nearest 2 mm Hg. The means of the right and left arm measurements were used as each participant’s systolic and diastolic blood pressures.

### Blood lead measurements

Blood samples for lead measurement were taken in special trace-metal-free tubes containing ethylenedi-aminetetra-acetic acid, and sent to ESA Laboratories, Inc. (Bedford, MA), for analysis. After room temperature digestion with nitric acid, the sample solution was centrifuged and the supernatant was poured into a sample cup. It was then analyzed by Zeeman background-correlated flameless atomic absorption (graphite furnace). The instrument was calibrated after every 21 samples with National Bureau of Standard Blood Lead Standards materials (Gaithersburg, MD). Ten percent of the samples were run in duplicate; at least 10% of the analyses were controls and 10% were blanks. A complete calibration check was made after the last specimen was analyzed. In tests on reference samples from the Centers for Disease Control and Prevention (Atlanta, GA), the coefficient of variation ranged from 8% for concentrations < 10 to 30 μg/dL, to 1% for higher concentrations. In comparison to a National Bureau of Standards (Gaithersburg, MD) target with a known blood lead concentration of 5.7 μg/dL, 24 repeated measurements conducted by ESA Labs using this method gave a mean ± SD of 5.3 ± 1.23 μg/dL.

### KXRF bone lead measurements

Bone lead measurements were performed from each participant’s mid-tibial shaft and patella with a KXRF instrument (ABIOMED Inc, Danvers, MA). The physical principles, technical specifications, validation, and quality control procedures of this (Burger et al. 1990; Hu et al. 1990, 1994) and other KXRF instruments (Jones et al. 1987; Somervaille et al. 1985) are described elsewhere. Because this instrument provides a continuous unbiased point estimate that oscillates around the true bone lead value, negative point estimates are sometimes produced when the true bone lead value is close to zero. An estimate of the uncertainty associated with each instrument, derived from a goodness-of-fit calculation of the spectrum curves and equivalent to a single standard deviation, is also provided. Although a minimum detectable limit calculation of twice this value has been proposed for interpreting an individual’s bone lead estimate (Gordon et al. 1993), retention of all point estimates makes better use of the data in epidemiologic studies (Kim et al. 1995). As a standard quality-control procedure of KXRF measurements, tibia and patella bone lead measurements with uncertainty estimates of > 10 μg/g and > 15 μg/g, respectively, of bone mineral were excluded. For our study, 30-min measurements were taken at the mid-shaft of the left tibia (representing cortical bone) and at the left patella (representing trabecular bone), after each region was washed with a 50% solution of isopropyl alcohol. The KXRF beam collimator was sited perpendicular to the bone surface for the tibia and 30 degrees in the lateral direction for the patella.

### Statistical analysis

We calculated univariate statistics and examined them for cases and noncases of IHD. We used chi-square or *t*-tests to assess the difference across cases and noncases. Blood and bone lead levels were log-transformed because their distributions were skewed. A value of 35 was added to tibia and patella lead levels before log-transformation (Kim et al. 1995; Kosnett et al. 1994).

We assessed the association between lead levels and risk for subsequent development of new IHD using Cox’s proportional hazards models. The follow-up period started at the time of baseline visit (after 1 September 1991) and lasted until the time of first IHD event or death from myocardial infarction, whichever occurred first. If the participant did not have an IHD event, the follow-up period ended on the date of last visit (before 31 December 2001) or 31 December 2001 (if the participant had a visit after 31 December 2001). Because only the year of IHD event was available, 31 December of the year in which the event occurred was used to calculate person-years for all incident cases.

We selected possible confounders on the basis of their biologic significance and information from previous studies. These covariates included age, body mass index, education, race, current smoking status, pack-years smoked, alcohol intake (grams per day) (Moller and Kristensen 1992; Pocock et al. 1988), history of diabetes mellitus and hypertension (Barzilay et al. 1998; Castelli et al. 1989), family history of hypertension, diastolic and systolic blood pressure, serum triglycerides, serum high-density lipids, and total serum cholesterol. Variables significant at the 0.10 level in univariate models were included in final multivariate models. Each of the log-transformed lead biomarker variables (blood lead, tibia lead, and patella lead) was then added separately into the multivariate models. We also analyzed blood lead as a categorical variable (≥ 5 μg/dL, ≥ 10 μg/dL, and ≥ 15 μg/dL) and bone lead in tertiles. To check for any residual or negative confounding, all covariates were again added, one at a time, in the final regression models. We performed a sensitivity analysis for all final regression models after excluding patients with diabetes mellitus (Barzilay et al. 1998; Castelli et al. 1989).

Statistical analysis was performed using SAS for UNIX (version 9.0; SAS Institute Inc., Cary, NC). The authors had full access to the data and take responsibility for its integrity. All authors have read and agree to the manuscript as written.

## Results

A comparison of participants included in our study with nonparticipants in the KXRF bone lead study, within the same time frame, revealed no significant differences with respect to age, race, body mass index, alcohol intake, smoking, a family history of hypertension, systolic and diastolic blood pressure, and a history of diabetes mellitus or hypertension (data not shown). A similar comparison of participants included in our study with those who did not return for a follow-up visit during our study time frame also yielded no significant differences between the two groups.

Of the 837 participants in our study, an IHD event occurred in 83 cases (70 nonfatal and 13 fatal). The mean age of noncases (65.9 ± 7.3 years) was similar to that of cases (67.5 ± 6.5 years). The distribution of other covariates—including known risk factors for IHD such as smoking, alcohol intake, systolic and diastolic blood pressures, a history of diabetes mellitus or hypertension, serum triglycerides, and total serum cholesterol—was also similar among cases and noncases (Table 1). However, the person-time contributed by noncases was significantly longer than cases, because cases were censored once an IHD event occurred.

The mean blood, tibia, and patella lead levels were higher in IHD cases than in non-cases. When blood lead level was examined as a categorical variable, the proportion of cases with a blood lead level ≥ 5 μg/dL was significantly higher than noncases. When bone lead was examined in tertiles, a higher proportion of cases were in the highest tertile of tibia and patella lead level compared with noncases (for tibia lead: 38.1% cases compared with 32.7% noncases; for patella lead: 49.2% cases compared with 30.8% noncases) (data not shown).

Age and serum high-density lipids were associated with IHD in multivariate Cox proportional hazards regression models such that the risk for IHD increased with increasing age and decreased with increasing serum high-density lipids (Table 2). When assessed as continuous variables, an increase in blood or bone lead level was associated with higher risk for an IHD event. As a categorical variable, blood lead level ≥ 5 μg/dL had a hazard ratio of 1.73 [95% confidence interval (CI), 1.05–2.87] for IHD compared with blood lead level < 5 μg/dL. A dose response was not noted when tibia and patella lead levels were analyzed in tertiles and quartiles.

The inclusion of other covariates known to be risk factors for coronary disease—such as body mass index, alcohol consumption, current smoking, pack-years, a diagnosis of diabetes, a diagnosis of hypertension, blood pressure, family history of hypertension, total serum cholesterol, and total serum triglycerides—in the final regression models did not alter our findings on the association between lead and IHD (data not shown). Our results were also similar when participants with diabetes were excluded from the analysis (data not shown).

The correlation between blood and bone lead levels was modest (correlation coefficient = 0.30 for tibia and blood lead, and 0.37 for patella and blood lead). As expected, tibia and patella lead levels were strongly correlated with each other (correlation coefficient = 0.78). When blood lead and one of the bone lead variables were assessed in regression models simultaneously, the individual effect estimates of blood and bone lead were only moderately attenuated. The hazard ratio for log blood lead was 1.24 (95% CI, 0.80–1.93) and for log patella lead was 2.62 (95% CI, 0.99–6.93) when these variables were assessed together in a multivariate model. Similarly, the hazards ratio for blood lead was 1.38 (95% CI, 0.89–2.13) and that for tibia lead was 1.55 (95% CI, 0.44–5.53) when these variables were included together in the model.

## Discussion

The relationship between biomarkers of long-term lead exposure and IHD has not been previously assessed. In a longitudinal study of 837 middle-aged and elderly men followed from 1 September 1991 through 31 December 2001, we found that the risk of future IHD increases significantly with increasing bone and blood lead levels, after adjusting for potential confounders.

The relationship of lead exposure with hypertension and increased blood pressure has been established in previous studies (Cheng et al. 2001; Harlan 1988; Hertz-Picciotto and Croft 1993; Hu et al. 1996a; Kopp et al. 1988; Martin et al. 2006; Moller and Kristensen 1992; Nash et al. 2003; Pirkle et al. 1985; Schwartz 1991, 1995). Furthermore, it has also been reported that higher blood lead levels lead to increased mortality from cardiovascular causes (Lustberg and Silbergeld 2002). However, only three previous investigations have assessed the association between blood lead levels and heart disease (Kromhout 1988; Moller and Kristensen 1992; Pocock et al. 1988). Pocock et al. (1988) followed 7,371 men 40–59 years of age in Britain for 6 years, to assess the relationship between blood lead levels at baseline and IHD. Although mean blood lead concentration was significantly higher in cases (0.786 μmole/L) than in non-cases (0.735 μmole/L), there was no evidence that blood lead was associated with IHD after controlling for potential confounders. Moller and Kristensen (1992) studied the risk of fatal and nonfatal coronary heart disease and cardiovascular disease in 1,050 participants after 14 years of follow-up. Their results were similar to those of Pocock et al. in that blood lead was associated with increased risk for coronary heart disease (relative hazard = 2.14; *p* = 0.003) and cardiovascular disease (relative hazard = 1.58; *p* = 0.05) in an unadjusted analysis; but the association disappeared when confounders were adjusted for. Another smaller study (*n* = 141) by Kromhout (1988) in the Netherlands found no association between blood lead and coronary heart disease in univariate and multivariate analysis. However, only 26 participants had coronary heart disease in their 8 years of follow-up data. A recent case report described a patient with angina (severe spontaneous chest pain with S-T elevation) who had a normal coronary angiogram and blood lead level of 33 μg/dL (Oneglia et al. 1998). The patient was chelated with EDTA, and described to be normal during follow-up. The authors hypothesized that lead exposure was possibly involved in endothelial dysfunction and coronary spasm in this case.

It is likely that previous studies (Kromhout 1988; Pocock et al. 1988), although suggestive of a relationship between lead exposure and heart disease, did not find an association in multivariate analysis because of differences in study population. Another likely reason is that blood lead was used as a biomarker for exposure. Lead accumulates in the skeleton, with a half-life of years to decades (Manton 1985; Rabinowitz et al. 1976). Bone is a repository for 90–95% of lead in adults (Barry and Mossman 1970; Saltzman et al. 1990; Schroeder and Tipton 1968). Previous studies have shown that bone lead levels remain elevated despite declines in blood lead. Therefore, bone lead may be the biomarker of choice for measurement of long-term lead exposure. Bone lead levels have been found to be better predictors than blood lead when assessing outcomes such as hypertension and cognitive declines in a number of recent studies (Cheng et al. 2001; Hu et al. 1996a; Schwartz et al. 2000; Weisskopf et al. 2004). There is evidence that lead is released from bone stores, especially during increased bone turnover (Rabinowitz 1991; Silbergeld 1991). This may contribute to increased blood lead in persons with increased bone lead or increased bone turnover.

Blood and bone lead were associated with increased risk for IHD in our study. Furthermore, the effect estimates of blood and bone lead were not attenuated when assessed simultaneously, suggesting that both contribute independently to IHD. It is unclear why tibia lead was not significantly associated with IHD, although the direction of association was consistent with our overall findings. The stronger association of patella lead with IHD is noteworthy in that the patella is composed of trabecular bone and is known to have higher turnover rates and contribute more to blood lead than the cortical bone represented by tibia lead (Hu et al. 1996b). Because bone lead may contribute to blood lead, particularly in our aging cohort, which has had greater historic environmental exposures and higher rates of bone resorption, the association of blood lead with IHD is plausible. It is also likely that persons in the general population with high blood lead levels have historically had higher levels of lead. In summary, blood lead level reflects acute exposure from circulating lead, whereas bone lead reflects chronic exposure as well as the major internal source of circulating blood lead. Both factors likely play a role in predicting risk for IHD. We suggest that future studies look at both blood and bone lead when assessing the risk for IHD from lead exposure.

The pathogenesis of the association between lead exposure and IHD can be explained by two mechanisms: One is mediation through increase in blood pressure, which has been previously associated with an increase in risk for ischemic and coronary heart disease (Khot et al. 2003; MacMahon et al. 1990; Tibblin et al. 1975; Wojtczak-Jaroszowa and Kubow 1989); and the other is by atherogenic process. Atherosclerosis can result from lead exposure by inhibition of cytochrome P-450, leading to accumulation of lipids in vessel walls. Lead exposure can also lead to inhibition of superoxide dismutase, an oxygen radical–scavenging enzyme, leading to an increase in serum lipid peroxide (Moller and Kristensen 1992; Wojtczak-Jaroszowa and Kubow 1989). Serum peroxide is a risk factor for vascular disease and thrombus formation.

Although lead levels have declined in the United States and other industrialized nations, low-level lead exposures still persist, and exposure from higher lead levels in the past is likely. Because the pathogenesis of IHD is chronic and takes years to develop, the public health implications of cumulative lifetime lead exposure in the general population are likely being currently realized and will continue in the near future.

Our study was limited by the unavailability of exact date of onset for the IHD event. Therefore, 31 December of the year of IHD diagnosis was used in person-time calculations. However, it is unlikely that this would lead to a differential bias by IHD status. Because our study population included only men and had very few minority participants, our results may not be generalized to races other than white or to women. Our study also had a limited number of IHD events. Therefore, residual confounding unaccounted for in our analysis is a possibility. This includes factors such as measures of socioeconomic status that are related to lead levels. A lower socioeconomic status may lead to inadequate health maintenance, thereby increasing the risk for IHD.

## Conclusion

In summary, we found that men with increased blood and bone lead levels were at an increased risk for future IHD. Low-level lead exposures in the recent past and higher past exposures may contribute to the increased risk for IHD. Although, the pathogenesis of IHD is multifactorial, lead exposure may be one of the risk factors for development of IHD.

## Figures and Tables

**Table 1:** Baseline characteristics of IHD cases and noncases, Normative Aging Study, 1991–2001.

	Noncases (*n* = 754)			Cases (nonfatal *n* = 70; fatal *n* = 13)		
Characteristic	Total^a^	No. (%)	Range	Total^a^	No. (%)	Range
Age (years)*						
< 60	754	162 (21.5)	—	83	10 (12.1)	—
60–69	754	378 (50.1)	—	83	48 (57.8)	—
≥ 70	754	214 (28.4)	—	83	25 (30.1)	—
Race**						
White	747	734 (98.3)	—	82	78 (95.1)	—
Black	747	13 (1.7)	—	82	4 (4.9)	—
Current smoker	754	60 (8.0)	—	83	4 (4.8)	—
Pack-years (among smokers)^b^	528	29.7 ± 23.7	0.10 to 145.5	61	34.0 ± 30.4	0.20 to 110.0
Body mass index (kg/meter^2^)^b^	749	28.0 ± 3.8	16.7 to 51.3	82	28.4 ± 3.8	19.6 to 41.5
Serum triglycerides (mg/dL)^b^	743	151.2 ± 93.9	24.0 to 978.0	83	146.5 ± 60.9	49.0 to 340.0
Total serum cholesterol (mg/dL)^b^	753	230.5 ± 38.7	130.0 to 438.0	83	232.7 ± 32.6	158.0 to 297.0
Serum high-density lipids (mg/dL)^b^*	730	49.4 ± 13.3	21 to 131	83	45.8 ± 10.3	21.0 to 85.0
Alcohol intake (gm/day)^b^	737	13.3 ± 17.5	0.0 to 104.1	80	11.2 ± 14.6	0.0 to 67.0
Systolic blood pressure (mm Hg)^b^	753	134.9 ± 17.0	91.0 to 215.0	83	136.4 ± 18.6	103.0 to 186.0
Diastolic blood pressure (mm Hg)^b^	753	82.0 ± 9.3	51.0 to 122.0	83	81.5 ± 11.3	56.0 to 110.0
Diabetes	754	81 (10.7)	—	83	9 (10.8)	—
Hypertension	754	341 (45.2)	—	83	42 (50.6)	—
Family history of hypertension	635	277 (43.6)	—	72	30 (41.7)	—
Person time (years)^b^*	754	6.9 ± 2.3	1.8 to 10.4	83	3.8 ± 2.7	0.08 to 10.7
Blood lead (μg/dL)^b^	738	6.2 ± 4.3	0.0 to 35.0	80	7.0 ± 3.8	1.0 to 20.0
Blood lead* tertiles						
< 5 μg/dL	738	306 (41.5)	—	64	22 (27.5)	—
5–9.9 μg/dL	738	329 (44.6)	—	64	43 (53.8)	—
≥ 10 μg/dL	738	103 (14.0)	—	64	15 (18.8)	—
Patella lead (μg/g)^b^*	487	30.6 ± 19.7	–10.0 to 165.0	63	36.8 ± 20.8	5.0 to 101.0
Patella lead (μg/g)^b^* tertiles						
Tertile 1	487	13.9 ± 4.9	–10.0 to 20.0	63	15.3 ± 4.3	5.0 to 19.0
Tertile 2	487	27.1 ± 4.1	21.0 to 34.0	63	25.7 ± 3.8	21.0 to 33.0
Tertile 3	487	52.5 ± 20.7	35.0 to 165.0	63	53.3 ± 17.3	35.0 to 101.0
Tibia lead (μg/g)^b^	486	21.4 ± 13.6	–3.0 to 126.0	63	24.2 ± 15.9	–5.0 to 75.0
Tibia lead (μg/g)^b^ tertiles						
Tertile 1	486	10.2 ± 3.8	–3.0 to 15.0	63	10.1 ± 5.3	–5.0 to 15.0
Tertile 2	486	19.1 ± 2.3	16.0 to 23.0	63	19.8 ± 2.2	16.0 to 23.0
Tertile 3	486	35.5 ± 14.4	24.0 to 126.0	63	39.5 ± 14.9	25.0 to 75.0

a Total *n* for the respective variable.

b Mean ± SD.

* *p* < 0.05 for cases versus noncases.

** *p* < 0.10 for cases versus noncases.

**Table 2:** Cox proportional hazards models for the association between biomarkers of lead level and IHD, Normative Aging Study, 1991–2001 [HR (95% CI)].

Covariate	Unadjusted	Model A (*n* = 787)	Model B (*n* = 787)	Model C (*n* = 532)	Model D (*n* = 531)
Age (years)					
< 60	Reference	Reference	Reference	Reference	Reference
60–69	2.18 (1.10–4.32)	2.43 (1.19–4.97)	2.45 (1.20–5.03)	1.67 (0.77–3.64)	1.71 (0.78–3.76)
≥ 70	2.44 (1.16–5.10)	2.52 (1.15–5.49)	2.57 (1.18–5.61)	2.01 (0.83–4.84)	2.22 (0.91–5.42)
Black race	2.38 (0.87–6.49)	1.84 (0.58–5.90)	1.71 (0.53–5.53)	1.99 (0.61–6.45)	2.14 (0.66–6.94)
Serum high-density lipids (mg/dL)	0.97 (0.96–0.99)	0.97 (0.95–0.99)	0.97 (0.95–0.99)	0.98 (0.96–1.00)	0.98 (0.96–1.00)
Blood lead level ≥ 5 μg/dL	1.64 (1.00–2.68)	1.73 (1.05–2.87)*	—	—	—
Blood lead level (μg/dL)^a^	1.40 (0.99–1.98)	—	1.45 (1.01–2.06)*	—	—
Patella lead level (μg/g)^a^	3.27 (1.41–7.58)	—	—	2.64 (1.09–6.37)*	—
Tibia lead level (μg/g)^a^	2.76 (0.94–8.12)	—	—	—	1.84 (0.57–5.90)**

HR, hazard ratio; CI, confidence interval. The hazard ratios and their statistical significance for blood and bone lead were similar when other potential confounders such as smoking, body mass index, alcohol consumption, blood pressure, family history of hypertension, and total serum cholesterol were included in the models.

a Logarithm of lead level.

* *p* = 0.05;

** *p* = 0.31.
